# Data management routines for reproducible research using the G-Node Python Client library

**DOI:** 10.3389/fninf.2014.00015

**Published:** 2014-03-05

**Authors:** Andrey Sobolev, Adrian Stoewer, Michael Pereira, Christian J. Kellner, Christian Garbers, Philipp L. Rautenberg, Thomas Wachtler

**Affiliations:** Department of Biology II, Ludwig-Maximilians-Universität MünchenPlanegg-Martinsried, Germany

**Keywords:** electrophysiology, data management, experimental workflow, neuroinformatics, python, neo, odml, web service

## Abstract

Structured, efficient, and secure storage of experimental data and associated meta-information constitutes one of the most pressing technical challenges in modern neuroscience, and does so particularly in electrophysiology. The German INCF Node aims to provide open-source solutions for this domain that support the scientific data management and analysis workflow, and thus facilitate future data access and reproducible research. G-Node provides a data management system, accessible through an application interface, that is based on a combination of standardized data representation and flexible data annotation to account for the variety of experimental paradigms in electrophysiology. The G-Node Python Library exposes these services to the Python environment, enabling researchers to organize and access their experimental data using their familiar tools while gaining the advantages that a centralized storage entails. The library provides powerful query features, including data slicing and selection by metadata, as well as fine-grained permission control for collaboration and data sharing. Here we demonstrate key actions in working with experimental neuroscience data, such as building a metadata structure, organizing recorded data in datasets, annotating data, or selecting data regions of interest, that can be automated to large degree using the library. Compliant with existing de-facto standards, the G-Node Python Library is compatible with many Python tools in the field of neurophysiology and thus enables seamless integration of data organization into the scientific data workflow.

## 1. Introduction

Recent advancements in technology and methodology have led to growing amounts of increasingly complex data recorded from various species, modalities, and levels of study. Annotation and organization of these data, which is not only important for reproducibility of results and re-use of data, but also essential for collaboration and data sharing, has become a challenging task. An important requirement for consistent organization of data is the availability of metadata that provide information about the experimental conditions and context in which the data were recorded, to enable meaningful analysis and comparison of results. This is especially important in neurophysiology, with its enormous variety of electrode configurations, types of signals recorded, species, and experimental paradigms. With advancing methodologies and increasing complexity of experimental paradigms, and consequently complexity and volume of data, it can become challenging to keep track of data even within a single lab and, for example, access data for re-use some time after the study was completed. When it comes to collaboration across labs, questions of data organization, data access and data sharing become even more critical. To help scientists deal with these challenges, the German INCF Node^1^ (G-Node) is developing software solutions consisting of services and tools for data access and data management in this field.

Several initiatives to support sharing of neurophysiology data have emerged in the past years. Among those are CRCNS.org^2^, CARMEN^3^, The INCF Japan Node (J-Node)^4^ Brain-Liner^5^, the recent INCF DataSpace^6^, and other projects. Most of the underlying solutions, however, were mainly designed to enable data exchange based on files and do not provide interfaces to operate with lower-level objects (data arrays, events, regions of interest etc.) or to extensively annotate these specific data objects. Furthermore, only a few of the current solutions were designed to support direct data access from the computational environment, in particular from the Python framework.

G-Node provides a data management system with functions for storage, organization, search, and sharing of data and metadata^7^ (Sobolev et al., in review) with various tools and enhancements^8^. These solutions are designed to support data collection and annotation within the data processing workflow and focus on data accessibility, enabling reproducible research. To make them widely usable and facilitate their use by integration with the scientist's established data handling routines, it is important to account for the variety of conventions and formats across labs. Python is a programming language that provides high flexibility, integrates well with other software, and is increasingly used in the neurosciences, including experimental labs. Here we present a Python library that exposes the functionality of the G-Node Data Platform to the Python user. Effortless integration with other tools (Garcia and Fourcaud-Trocmé, 2009; Davison et al., 2013; Pröpper and Obermayer, 2013) is enabled by using conventions already established in this field, such as the Neo common data model for electrophysiological data (Garcia et al., 2014) and the odML format for metadata (Grewe et al., 2011).

## 2. Approach

Goal of the G-Node Data Platform is to provide services and tools for organization and unified access to experimental data and metadata collected at different times or by different lab members or collaborators, to facilitate reproducible research and re-use of data. The solution builds on existing standards and software tools for easy integration with the researcher's established scientific data analysis routines.

### 2.1. Design principles

#### 2.1.1. Server-Client architecture

The G-Node Data Platform provides a storage and management system for scientific data, accessible through a network API^9^. Client tools enable accessing data and functions from different platforms, like Python or Matlab. Scientists can use the G-Node Data Platform server for remote data storage and data sharing, or install a local server instance for use in the lab. Having a centralized storage unifies data management routines within the lab and brings experimental recordings to a common format. If an experimentalist leaves the lab, recorded data stays available and accessible. A central data service introduces accessibility and location independence via remote network access, and provides a single way of data and metadata handling even for collaborators from other locations. Clients libraries, including the G-Node Python Library described in this manuscript, allow direct access to the experimental results from the local computational environment. This makes it easier to integrate into existing data analysis or modeling workflows.

Backend and interface of the G-Node Data Platform will be described in detail in another paper (Sobolev et al., in review). Here we briefly summarize the design principles and then focus on the functionality as exposed by the G-Node Python Library to the Python user.

#### 2.1.2. Data model

**Figure 1:**
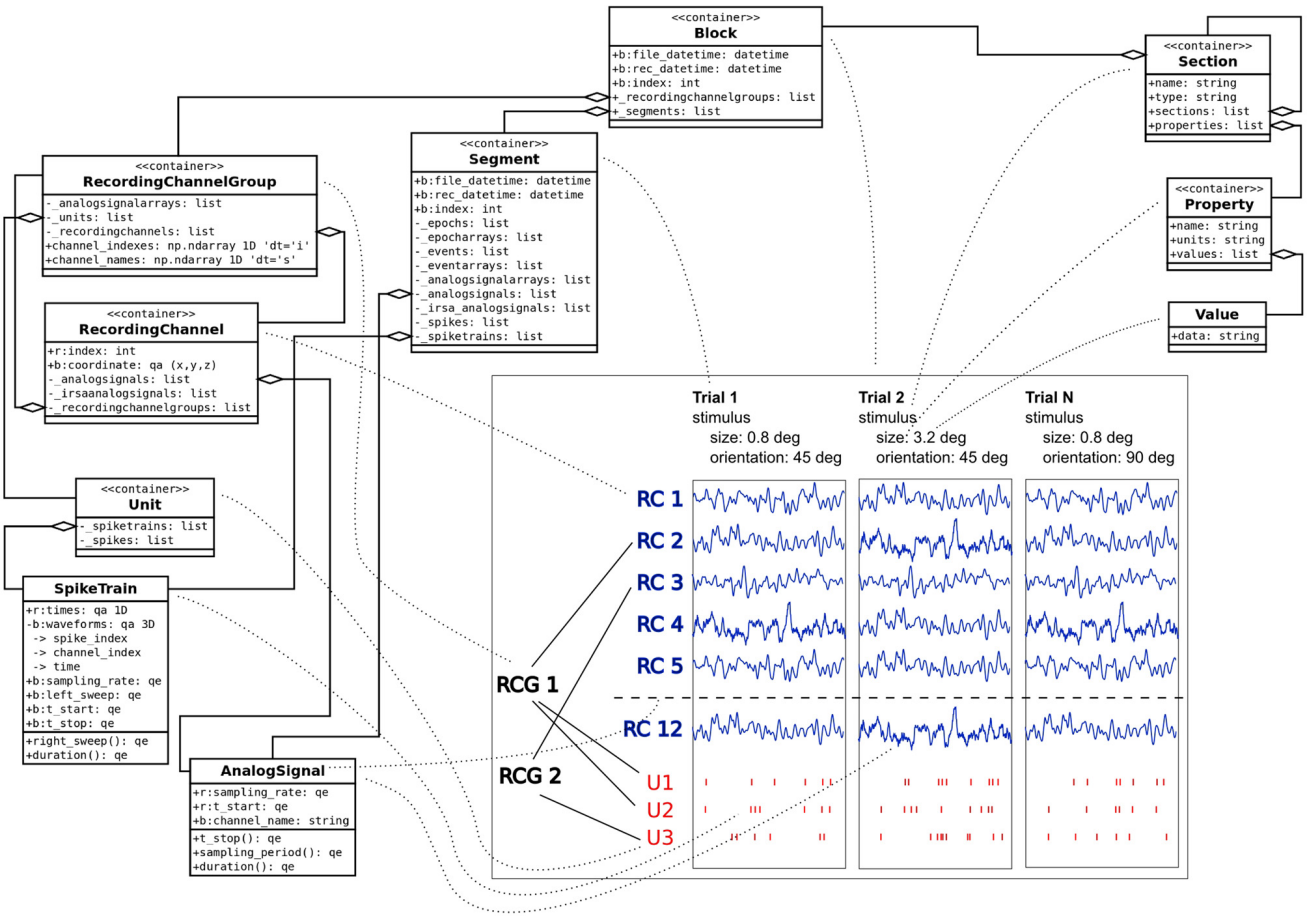
**Data model combining Neo and odML objects with key model classes used to describe experimental data and metadata.** Traces in the center of the plot illustrate experimental time segments (Trials 1, 2, …, N) containing LFP traces taken from corresponging channels (RC1, RC2, …, RC12). Each time segment contains also spike trains for every identified unit (U1, …, U3), which in turn is connected to recording channels via channel groups (RCG1, RCG2). Dotted lines denote connections between classes and the data they represent. Note that for clarity not all supported objects and attributes are shown on the figure.

G-Node Data Platform and G-Node Python Library build on tools, standards and conventions established in the field of electrophysiology. To address the need of facilitating standardized data access and at the same time accounting for the variety of experimental approaches in this domain, the approach is based on combining a standardized data model with a flexible and extensible metadata format (Figure 1).

The representation of recorded data follows the data structure defined by the Neo object model (Garcia et al., 2014). Neo is a Python library for electrophysiological data that also supports reading a wide range of neurophysiology file formats (Spike2, NeuroExplorer, AlphaOmega, Axon, Blackrock etc.). It implements a hierarchical data model to represent electrophysiological data entities with their relationships and minimal metadata (e.g., units, dimensions etc.). A typical experimental data representaton is a dataset (*Block* in Neo) containing several experimental trials (*Segments*), each having recorded time series signals (*AnalogSignals*), spike event data (*SpikeTrains*) and stimulus event times as *Events*. A dataset (*Block*) usually also contains information about grouping of channels (*Recording Channel Groups, Recording Channels*) to indicate spatial position and arrangement, and assignment of spike trains to single or multi units (*Units*). Neo is currently used by different electrophysiology labs and initiatives (see Garcia et al., 2014). The representation of data by the G-Node Python Library is based on the Neo Python library,^10^ and thus enables seamless integration with other software that uses the Neo objects.

Metadata are organized according to the odML data model (Grewe et al., 2011). odML is an open, flexible and easy to use format to organize metadata as key-value pairs (odML *Properties*) organized in a hierarchical structure (by odML *Sections*). *Sections* are used to meaningfully group *Properties* according to experimental aspects (Subject, Preparation, Stimulus, Hardware Settings etc.). odML *Sections* can be nested, enabling a flexible way to organize experimental metadata in a hierarchy that reflects the structure of the experiment. In addition, odML provides terminologies^11^ for commonly used sets of experimental descriptors, such as hardware properties, amplifier settings, stimulus parameters and many others, which can be used to achieve a standardized description of the experimental context (Grewe et al., 2011).

The G-Node Data Platform combines these structures into an integrated data representation with the possibility to link between recorded data and metadata. This enables comprehensive organization of data from any electrophysiological experiment and unified data access that facilitates data analysis.

### 2.2. Implementation

#### 2.2.1. Functional scope

The G-Node Python Library implements tools that operate on the local workstation in a native Python environment. It mainly consists of functions, designed to help maintaining experimental structures locally and periodically synchronize required datasets with the central storage. It provides a useful interface to access previously stored experimental datasets, search across all experimental entities, download any particular dataset, single spike trains or time series, and represent them in native Python objects.

Locally, the G-Node Python Library implements an interface to connect Neo and odML objects in a flexible way and to store annotated structures on disk. When the dataset is complete, data together with experimental annotations can be submitted to the central data storage and later be opened for access by particular collaborators or stakeholders. This is done by a set of G-Node Python Library functions that allow to manage permissions for any given object relative to a single user, several users or all user accounts. This fine-grained data access is discussed in more detail in the next sections.

#### 2.2.2. Technical design

The G-Node Python Library is written in pure Python and uses python-neo^12^ and python-odml^13^ as libraries to represent key model objects, requests^14^ library for HTTP transactions, appdirs^15^ to access local temporary and cache folders, requests-futures^16^ for asynchronous HTTP requests and h5py^17^ to handle array data stored in HDF5 format. Connection to the central data platform is done via REST (Fielding and Taylor, 2002) implemented over HTTP. The formats for data transfer are defined by the G-Node Data Platform. In particular, HDF5^18^ is used for data arrays and JSON^19^ for other objects, attributes or relationships. The library implements a local cache backend for transient storage of downloaded or newly created objects. A standard Python testing suite is included in the distribution.

The library implements lazy-loaded relationships, which allows accessing an object without fetching related objects. For some lightweight metadata analysis it is practical to use such relationships, as the download happens only when related objects are actually accessed. This kind of data access significantly reduces communication time and increases search and processing speed. However, it is also possible to download objects with all related array data and relationships at once, and have a complete dataset structure locally ready to be used in further computations.

Importantly, all output objects are real Neo or odML python objects as defined by python-neo and python-odml. This enables direct integration of the library with the existing custom scripts that already use these libraries.

## 3. Results

Here we present several use case examples of the G-Node Python Library to illustrate its application on real experimental data. We focus on aspects of the library that provide benefits to the scientific workflow within the whole experimental lifecycle, from the experiment planning stage to the stage of data analysis.

The G-Node Python Library is freely available at the G-Node github page^20^. Related documentation can be compiled locally with sphinx^21^ or accessed online at the project page^22^. For proper operation, the library requires a server part to be available. A demo environment^23^ is provided by the G-Node, which can be accessed for testing or introductory purposes without user account registration or local server installation.

All examples assume that the library is already installed and configured^24^. Importing the required modules and establishing the server connection is done with only a few lines of code: 
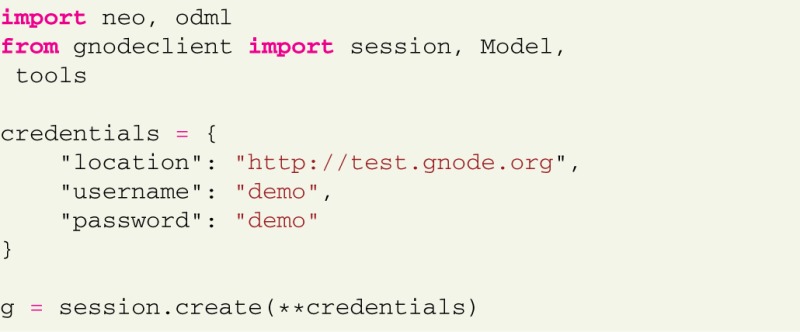
 The *session* class handles interaction with the remote server, the *Model* class contains model definitions, supported by the G-Node Python Library. The *tools* module is an additional collection of supplementing functions that add a layer of convenience on top of the primary G-Node Python Library functions. This is useful for combining frequently used functions or performing operations on multiple objects within a data structure. Some examples are given below.

For illustration, we consider a typical experimental study in which responses from neurons in the visual cortex of macaque monkeys are recorded (e.g., Teichert et al., 2007). Detailed description of the experiments is omitted; instead, only the key data and metadata entities are described as relevant for the current paper. In this example, we assume neural responses recorded with an array of electrodes. Local field potential (LFP) signals were obtained by hardware bandpass filtering, and spike trains by online spike sorting. The experiment consisted of different trials with stimulus parameters varying from trial to trial. Visual stimuli were gratings varying in size, orientation, and spatial frequency, presented one at a time. An experimental dataset is represented as a Neo *Block* having experimental trials represented as Neo *Segments*. Each trial (*Segment*) contains corresponding raw LFP data as *AnalogSignals* and sorted neural event data as *SpikeTrains*. Neo *RecordingChannels* are used to group signals recorded from the same electrode, Neo *Units* to group neural events triggered by the same source.

### 3.1. Consistent structure for efficient access to electrophysiological data

Here we show how experimental data, like LFP signals and spike trains, can be stored and accessed using the G-Node Python Library. The experimental data structure can be well accommodated by the Neo data model. To store the entire dataset recorded in one experimental session, a Neo “block” is created: 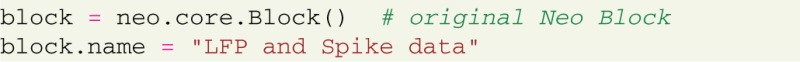 Then Neo objects for the data corresponding to the different trials and channels need to be created and connected to the block, including analog signals, spike trains, and units linked to the spike train objects and recording channels.

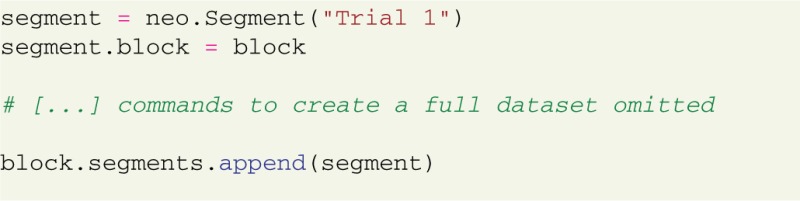



As these operations mainly use the standard python-neo library interface (see Garcia et al., 2014), the python code that creates the appropriate structure is omitted here. For illustration, a schematic figure of the current dataset (Figure 1) is provided.

Once the full data structure is defined, the *upload neo structure* function from the G-Node Python Library *tools* module is used to save all the data to the server: 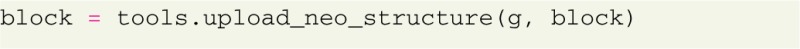 This operation submits the whole block with all connected recording channels and time segments, including related analog signals and spiketrains. After submission, data on the server can be accessed by type (e.g., time segment, analog signal) with filters^25^ using model attributes. For instance, according to the Neo model, analog signals have the sampling rate as an attribute. The following query requests analog signals with a certain sampling rate: 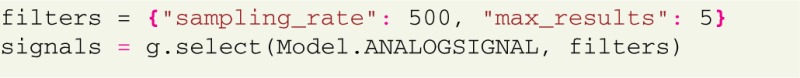 The “select” function of the G-Node Python Library accepts, as a second parameter, filters in a Python “dict” object.

Structured data can be accessed by spatial (e.g., *Recording Channel*), temporal (*Segment*), or source (*Unit*) criteria. The following request finds a certain recording channel and fetches all data coming from it: 
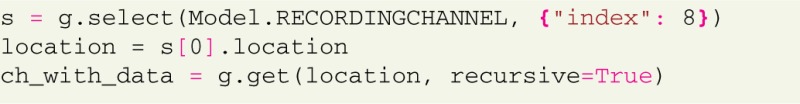
 Here the “select” function is used to query recording channel objects having “index” attribute set to 8. Every object, fetched from the server, has a “location” attribute which allows the library to determine the corresponding remote entity of the object. Then the “get” function allows to request the first channel from the previous selection with all related data recursively (analog signals, spike trains).

Another request finds a certain unit (in this example, a neuron given number 3) and fetches all spike trains detected from it: 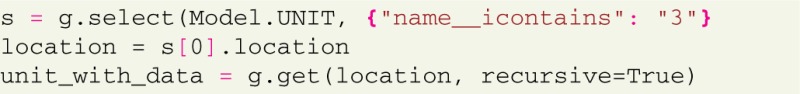


### 3.2. Collecting experimental metadata

For the organization of metadata, the G-Node Python Library provides an interface to the python-odml^26^ library, so that odML objects can be natively manipulated and stored to the central storage. odML terminologies can be loaded directly from the odML repository: 
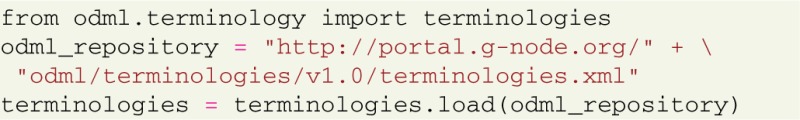
 Terminologies can be used as templates to describe certain parts of the experimental protocol. Among basic terminologies are templates for experiment, dataset, electrode, hardware configuration, cell etc^27^. These terminologies can be accessed as a Python “list” or “dict” as python-odml objects, and can be cloned to be used to annotate the current dataset: 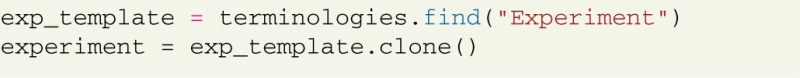 To describe the experiment, appropriate values are assigned to the properties: 
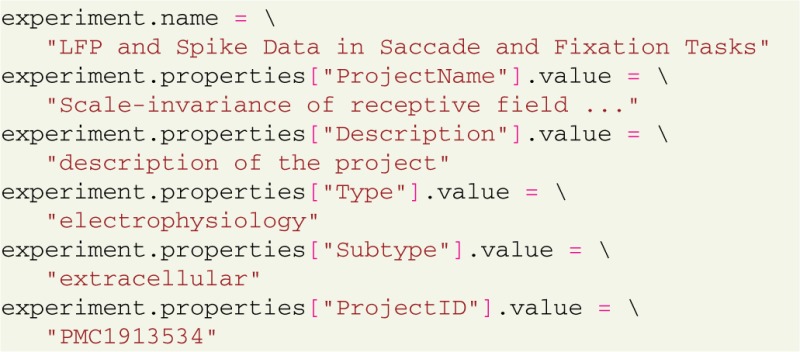
 Additional properties can be introduced as needed (Grewe et al., 2011). For example, stimulus parameters can be documented using custom odML section with custom properties: 
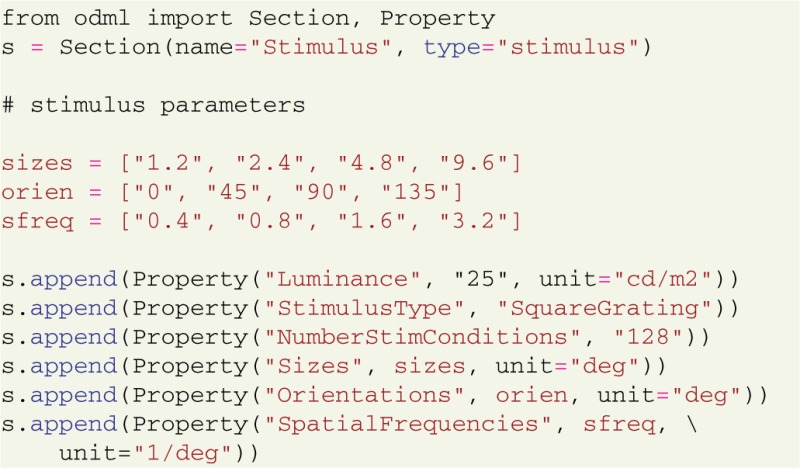
 Note that these assignments can be easily automatized if the parameters are available from the stimulation software or configuration files. Furthermore, if the parameters are stored by the software in odML format (Grewe et al., 2011), instead of creating metadata objects in Python, odML metadata structures can be read directly from files using the standard odML library. The odML format allows nested sections to capture the logical strucuture of the experiment. For example, a stimulus can be defined as part of an experiment: 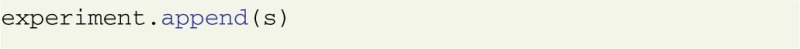 This tree-like structure can be saved with the G-Node Python Library: 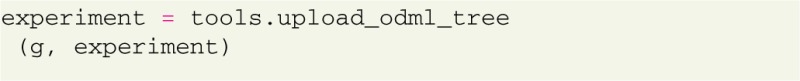 After submission, data and metadata stored on the server can be accessed in various ways. Metadata can not only be accessed as Python objects, using the G-Node Python Library, but also with Matlab, using the G-Node Matlab Toolbox^28^. Additionally, it can be browsed via a web interface^29^ or by custom software via the API.

As for the recorded data, the G-Node Python Library allows searching for metadata of a particular type, using different filters that can be applied for object attributes: 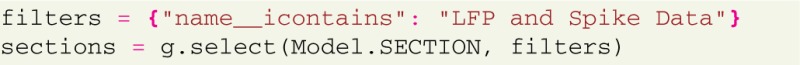 For complex experiments, the entire tree of metadata subsections can be very large. Therefore, the “select” function does not return the whole tree, instead it returns only the top level section objects with lazy-loaded relationship attributes, which will fetch related objects at the moment when they are first accessed. If the user wants to download the entire tree, it can be fetched with the “get” function with “recursive” parameter: 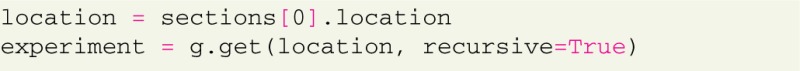 If another, similar experiment is performed, the metadata tree can simply be cloned and only the metadata that have changed updated. This is highly convenient and saves the time of re-entering parameters that stay the same across a series of experiments.

### 3.3. Connecting data and metadata

To meaningfully annotate data by metadata, the G-Node Python Library allows to connect datasets with the metadata: 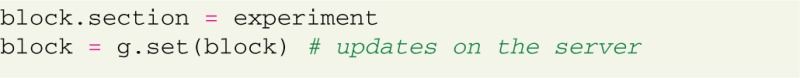 Note that an association between objects can only be set on one side of the one-to-many relationship. In this case a section can have many blocks, thus the block has to be changed to establish a connection. This constraint is imposed by the current Neo library and is expected to disappear in a future release. To work around potential limitations, the functions provided in the G-Node Python Library *tools* module can be used to conveniently create and upload data structures.

Additionally, the G-Node Python Library allows to connect data and metadata to indicate certain specific attributes for any of the Neo-type objects. A typical use case for this kind of data annotation is to specify which stimulus was applied in each trial of the experiment. This connection is done using the “metadata” attribute that uses existing metadata properties and values to “tag” a number of data-type objects: 
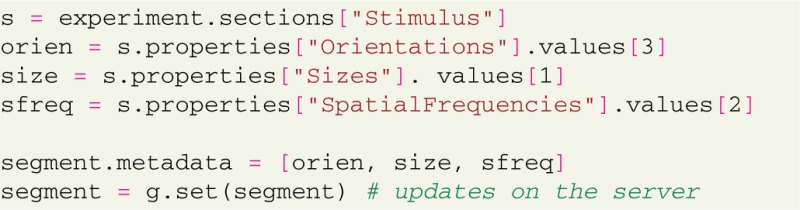
 Such assignments can easily be automatized if the parameters used in each trial can be obtained in machine-readable form from the software controling the experiment.

### 3.4. Data access from different angles

**Figure 2:**
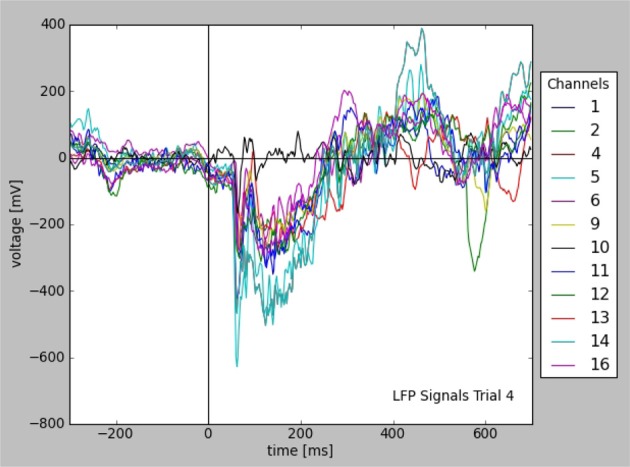
**Plot of LFP responses from a trial that was selected for a given stimulus configuration (see text).** Note that the information used for axes, labels, and legend was taken from the stored data and metadata directly.

Proper annotation brings more consistency in data and metadata, and allows to select data by metadata in various ways. For example, for data analysis it is often necessary to select all data recorded under the same experimental conditions. The following example selects all LFP data across all trials with a certain stimulus properties: 
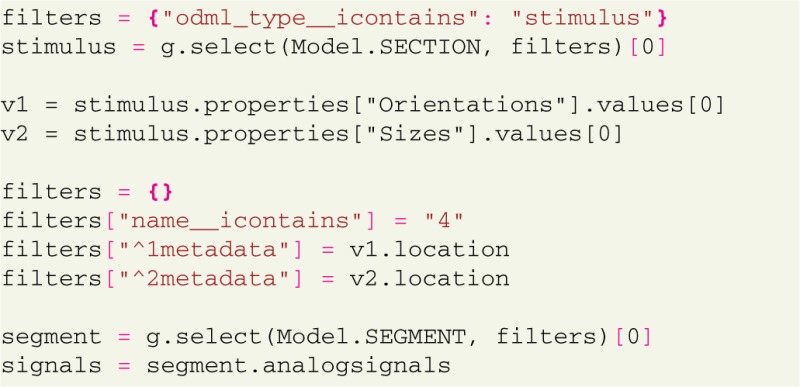
 In this example we select a section describing the stimulus and use stimulus parameter values to build a required filter. This filter is then used to query the trials where this particular stimulus was applied. This type of query makes it straightforward, for instance, to compute averages across trials for a certain stimulus configuration, 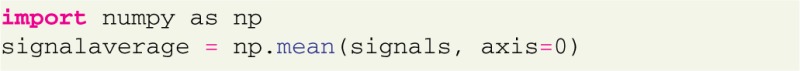 or to plot the actual LFP traces for visualization: 
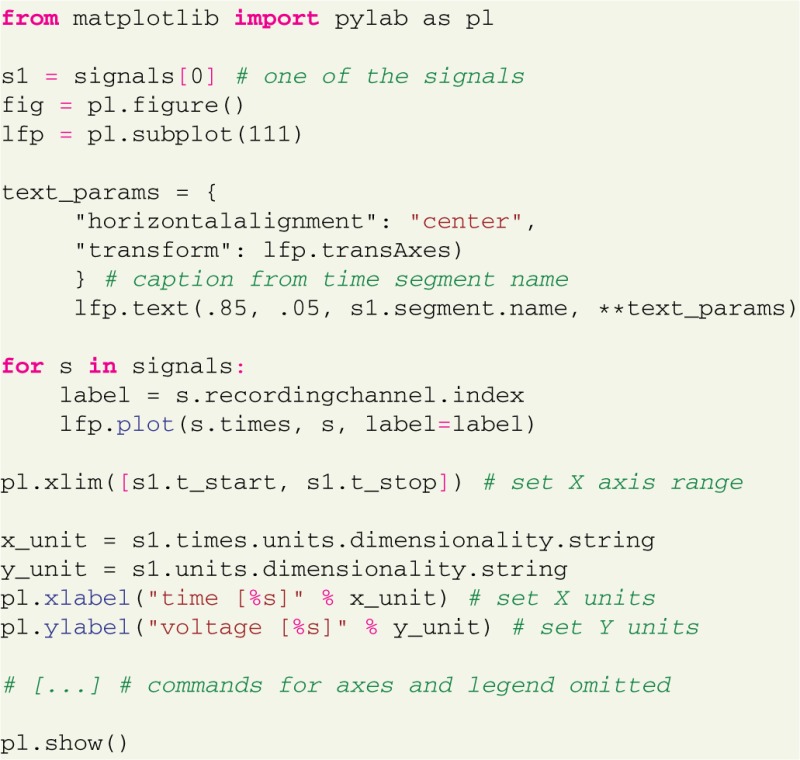
 Figure 2 illustrates the resulting plot. Note that the availability of metadata together with data immediately enables meaningful labeling of the axes without having to collect further information from files or hand-written documentation. Aside from being convenient and time efficient, this integration also offers enormous potential for automated analysis and facilitates reproducible research.

### 3.5. Integration into experimental workflow

One of the key objectives is easy integration into existing scientific environments or data processing workflows. This often requires access to data stored in proprietary formats. For this purpose, the G-Node Python Library uses Neo I/O, Python modules that can access a variety of data formats from different aquisition systems (Garcia et al., 2014).

Compatibility with Neo enables object representation ready for direct use in modeling or data analysis. Electrophysiology data objects and their attributes are represented using standard Python numerical packages, like “numpy”^30^, “scipy”^31^, and “quantities”^32^. This implies that every data object is a numpy array or has a numpy-based data attribute, with units implemented as Python “quantities.” Data access queries with the G-Node Python Library return Neo python objects that are ready to be included into analysis scripts, simulation or modeling programs, making data access more native and “pythonic.”

### 3.6. Implementation features

#### 3.6.1. Data caching

The library uses a local cache backend to store transient data, downloaded from the server. All downloaded data is available for offline use. Any downloaded data object will not be downloaded again if selected by a subsequent query, unless the object has changed or the cache is cleared explicitly.

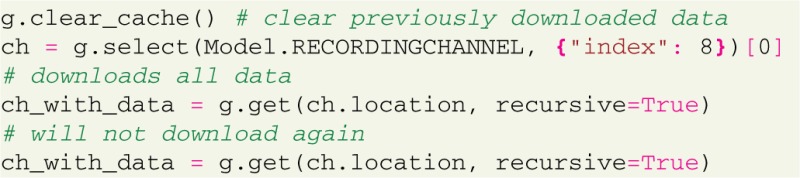



Objects stored in the cache are permanently available between sessions, unless the *clear cache* function is called. This significantly increases performance when several computations on the same dataset are required. In case an object is changed on the server, changes can be explicitly fetched by using a “refresh” parameter: 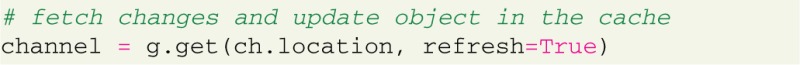


#### 3.6.2. Permissions

The G-Node Python Library allows fine-grained managing of permissions to access the data objects. Access to every object on the server can be opened for a particular user by the original object owner. This is particularly useful to support collaborative work and sharing of data.

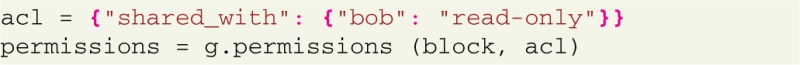



Managing permissions may usually be more conveniently done through the web interface of the G-Node Data Platform. Nevertheless, these functions are also available in the library at the Python level.

## 4. Discussion

The G-Node Python Library offers a combined local and remote way of handling electrophysiological data. To replace the usual way of copying data between hard disks and shared folders, G-Node provides a central storage where scientists can organize experimental data together with metadata. This approach of unified data and metadata management is a key to achieve reproducibility and has several advantages, especially in the long term. It is easier to maintain reproducibility when data are hosted at a central storage, either in the lab or at a remote server, even years after the study was done. Data kept at a single, accessible place can be easily opened for collaborators. Keeping data and metadata together in a standardized format requires less time to understand the data, thus finding and accessing the desired data as well as performing appropriate analyses is strongly facilitated. Furthermore, a standardized data representation makes it straightforward to apply analysis or visualization tools, or to compare the data with other results from experimental or simulation studies.

### 4.1. Extensions and future developments

#### High-level interface

For efficient use of the G-Node Python Library the neuroscientist has to become familiar with the Neo and odML concepts and data models. Adapting data and metadata handling and formats to the Neo and odML standards may require some efforts. However, the long-term benefits of interoperability and reproducibility that the use of common formats and interfaces achieves will outweigh these initial costs for many labs. To further lower the entrance barrier we started to develop high-level functions that allow to automate certain operations and provide patterns for creation of common data structures. Some of these functions are already available in the *tools* module of the G-Node Python Library. To increase the coverage of use cases, we encourage users to contribute their own custom functions.

#### Search and query

One of the key advantages of the G-Node Python Library is the potential to have all the information about a dataset available for easy search and efficient querying of data. Currently the search implemented in the G-Node Python Library is still limited to basic functions. While even with this limitation the availability of data and metadata for search brings a huge advantage, the full potential of this approach will only be exploited with more advanced search capabilities including relationships between object types and options for refined queries across the metadata. These extensions are currently in development.

#### Working offline

To minimize data transmission, as well as for practical reasons, a permanent connection to the server is not always desired. In some situations it is more suitable to work locally on the data or metadata and, when complete, submit appropriate structures to the server. Therefore a local storage management to save and access new data and metadata objects in the cache before syncing to the server is being developed and will be included in the next version of the G-Node Python Library. Several functions built on top of the main library interface are already available via the *tools* module, including functions that automatically resolve object relations and help to upload odML and Neo hierarchies recursively.

#### Integration with other Python tools

We are aiming at an even closer integration with other Python tools. The compatibility with the Neo data model makes it straightforward to combine the G-Node Python Library with other tools that use this data model. A pilot integration with the Spyke Viewer (Pröpper and Obermayer, 2013) is currently under development that will allow applying analysis scripts with Spyke Viewer directly on the data accessed via the G-Node Python Library. We are also developing a specific input/output module for the Neo package that supports reading and writing data to the G-Node Data Platform using the G-Node Python Library, so that every software using Neo can access data not only from data files but gains all the data management benefits of the G-Node Data Platform.

#### Standards and extension to other domains

The G-Node Python Library is built on a combination of existing formats with a focus on electrophysiology data. However, the same design principles can be easily applied to other domains. We plan extensions of the data objects to support imaging and morphological data. This will allow common organization of these multiple data types, which is also important for data obtained in research projects that employ multiple methods. Likewise, data objects specifically supporting analysis results will be implemented. The Standards for Data Sharing Program of the INCF is currently developing standards for formats and data structures for both the field of electrophysiology^33^ and the field of neuroimaging^34^. The G-Node Python Library will adopt those standards as they are released.

### Conflict of interest statement

The authors declare that the research was conducted in the absence of any commercial or financial relationships that could be construed as a potential conflict of interest.

## Footnotes

^1^www.g-node.org

^2^crcns.org

^3^www.carmen.org.uk

^4^www.neuroinf.jp

^5^brainliner.jp

^6^www.incf.org/resources/data-space

^7^github.com/G-Node/g-node-portal

^8^github.com/G-Node/

^9^g-node.github.io/g-node-portal/

^10^neuralensemble.org/neo

^11^www.g-node.org/projects/odml/terminologies

^12^github.com/NeuralEnsemble/python-neo

^13^github.com/G-Node/python-odml

^14^www.python-requests.org/

^15^pypi.python.org/pypi/appdirs

^16^github.com/ross/requests-futures

^17^www.h5py.org/

^18^www.hdfgroup.org/HDF5

^19^www.json.org

^20^github.com/G-Node/python-gnode-client

^21^sphinx-doc.org/

^22^g-node.github.io/python-gnode-client

^23^test.g-node.org

^24^g-node.github.io/python-gnode-client/install.html

^25^g-node.github.io/g-node-portal/key_functions/data_api/query.html

^26^github.com/G-Node/python-odml

^27^www.g-node.org/projects/odml/terminologies

^28^github.com/G-Node/gnode-client-matlab

^29^www.g-node.org/data/

^30^www.numpy.org

^31^scipy.org/scipylib/index.html

^32^pypi.python.org/pypi/quantities

^33^www.incf.org/programs/datasharing/electrophysiology-task-force

^34^www.incf.org/programs/datasharing/neuroimaging-task-force
